# Associations between male infertility and ancestry in South Americans: a case control study

**DOI:** 10.1186/s12881-017-0438-z

**Published:** 2017-07-26

**Authors:** Maria Fernanda Skowronek, Tatiana Velazquez, Patricia Mut, Gonzalo Figueiro, Monica Sans, Bernardo Bertoni, Rossana Sapiro

**Affiliations:** 1 Departamento de Histología y Embriología, Facultad de Medicina UDELAR, Montevideo, Uruguay; 2 Departamento de Genética, Facultad de Medicina UDELAR, Montevideo, Uruguay; 3 Departamento de Antropología, Facultad de Humanidades y Ciencias de la Educación, UDELAR, Montevideo, Uruguay

**Keywords:** Male infertility, Haplogroup, Semen parameters, Ancestry

## Abstract

**Background:**

Infertility affects 15% of human couples, with men being responsible in approximately 50% of cases. Moreover, the aetiology of male factor infertility is poorly understood. The majority of male factor infertility remains idiopathic and potentially genetic in origin. The association of the Y chromosome and mitochondrial haplogroups with male infertility has been previously reported. This association differs between studied populations and their geographical distributions. These effects have been only rarely analysed in mixed populations, such as South Americans.

**Methods:**

In this study, we analysed the contributions of the Y chromosome and mitochondrial haplogroups to male infertility in a mixed population. A case control study was conducted. Regular PCR and high-resolution-melting-real-time PCR were performed to type haplogroups from fertile and infertile men.

The sperm parameters from infertile men were compared in each haplogroup by logistic regression analysis and ANOVA.

**Results:**

The genotyping confirmed the known admixture characteristic of the Uruguayan population. The European paternal contribution was higher than the maternal contribution in both fertile and infertile men. Neither maternal nor paternal ancestry presented differences between the cases and controls. Men belonging to the Y chromosome haplogroup F(xK) more frequently presented with an abnormal sperm morphology than men from other haplogroups. The sperm parameters were not associated with the mitochondrial haplogroups.

**Conclusions:**

The data presented in this study showed an association between male infertility and ancestry in the Uruguayan population.

Specifically, abnormal sperm morphology was associated with the Y chromosome haplogroup F(xK). Since the Y chromosome lacks recombination, these data suggest that some genes that determine sperm morphology might be inherited in blocks with the region that determines specific haplogroups. However, the possible association between the Y chromosome haplogroup F(xK) and sperm morphology requires further confirmatory testing. Data linking infertility with ancestry are needed to establish the possible causes of infertility and define male populations susceptible to infertility. Whether the admixed characteristics of the Uruguayan population exert any pressure on male fertility potential must be further investigated.

**Electronic supplementary material:**

The online version of this article (doi:10.1186/s12881-017-0438-z) contains supplementary material, which is available to authorized users.

## Background

Infertility, defined as 1 year of unprotected intercourse without conception, affects approximately 15% of human couples, with men being responsible in approximately 50% of cases [1]. The aetiology of male infertility is poorly understood [2]. While some infertility cases can be explained by chromosomal abnormalities, endocrine disruptions, developmental abnormalities or environmental insults, a great percentage remains idiopathic and potentially genetic in origin [3–5].

Since the Y chromosome carries male factor determination, like SRY and other genes involved in male fertility, research has been especially focused on the analysis of the structure of this chromosome, looking for genetic associations with male infertility [6–8]. The structure of the Y chromosome includes a group of Y single-nucleotide polymorphisms in the male-specific region or MSY (male-specific Y). These polymorphisms present low rates of mutation, making them appropriate for identifying stable paternal lineages [9]. These polymorphic markers define monophyletic groups of the Y chromosome, or haplogroups. Several attempts have been undertaken to establish a classification and nomenclature system for defining Y haplogroups, e.g., the Y Chromosome Consortium (YCC). The YCC classifies haplogroups from A to T and further subdivides them according to the presence/absence of various biallelic markers [9, 10]. Now, the proposed classificatory system is being periodically revised by the International Society of Genetic Genealogy (ISOGG) https://isogg.org/tree/.

Due to a lack of recombination, Y polymorphic markers effectively tag the entire length of the male-specific region of the Y chromosome. Ninety-five percent of the length of the human Y chromosome is inherited as a single block in linkage from father to male offspring as a haploid entity. It is because of its particular properties (paternally inherited, lack of recombination and abundance of genetic polymorphisms) that the Y chromosome has been used as a tool for investigating recent human evolution from a male perspective [11]. Y chromosomal polymorphisms have also been used to study male-specific (spermatogenic failure, testis and prostate cancer) and prevalently male-associated (hypertension, autism) diseases [12, 13]. A limited number of studies have investigated the possible association of Y chromosome haplogroups with a particular infertile phenotype, and the contributions of predisposing factors or genetic background in causing spermatogenic failure remain debated. For example, Krausz et al. [14] identified a European haplogroup K associated with reduced sperm count and raised the possibility that selection might indeed be active on the Y chromosome in Danish males. Previdere et al. [15] found a significant difference in Y haplogroup distribution between infertile males and healthy controls among Italians, which could also be explained by the geographical origins of the subjects. In Asian populations, Y haplogroup K might bear a risk factor of male infertility [16], while studies in Japanese men have had inconsistent results. Carvalho et al. [17] did not find any association between haplogroups and infertility, but Y chromosome haplogroup D2 lineage was associated with azoospermia in Japanese males [18, 19].

Considering that each individual is a mix of two genomes, both maternal and paternal genetic background could contribute to the infertile phenotype. The geographical origin of a population can significantly impact sequence polymorphisms within its mitochondrial DNA (mtDNA), as well as Y chromosome haplogroups, which is a consequence of the special features of mtDNA, including matrilineal inheritance, lack of recombination, high copy number, and a higher evolutionary turnover rate, compared with nuclear DNA. mtDNA sequence variation evolved as a result of the sequential accumulation of mutations along maternally inherited lineages, which can be represented in a tree reflecting the phylogenetic relationships of known mtDNA variants that create clusters of related mtDNA haplogroups [20]. mtDNA haplogroups have been associated with disease susceptibility [21], such as to Alzheimer’s disease [22], Parkinson’s disease [23] and severe sepsis [24]. Several studies have also investigated mtDNA and male subfertile or infertile phenotypes [25–27]. The mitochondrial genome encodes 13 oxidative phosphorylation (OXPHOS) subunits and is essential for the production of adenosine triphosphate [28], which is vital for sperm motility [29]. Ruiz-Pesini et al. [26] reported the most famous studies of mtDNA haplotype variation in sperm motility. The authors showed that haplogroup H was underrepresented, and haplogroup T was overrepresented in men with low numbers of motile sperm in their ejaculate. However, other studies failed to identify an association between mtDNA and either sperm motility [30–32] or cellular bioenergetic parameters [33].

Redmon et al. found significant differences in semen parameters among men of different ethnicities [34]. There appear to be few data regarding semen parameters and ethnicity, particularly in males of African or Hispanic ancestry [34]. Moreover, these studies, as well as those that determined World Health Organization (WHO) reference ranges [35], were based on men living in Australia, Europe and North America. Data linking semen parameters with ethnic differences among people living outside these continents are lacking but necessary to establish the possible causes of infertility.

In particular, there have been very few studies of mixed populations. The non-European genetic contribution to the population of Uruguay has been estimated using autosomal markers as ~10% Native American and ~6% African [36]. However, maternal lineages assessed using mtDNA revealed a Native American ancestral proportion of 62% in the north and 20% in the south (mean value for the country ~ 34%), while the African contribution varies between 8% and 21% [37–42]. Based on previous results [37, 42], as well as sex-biased patterns in the process of gene flow in other Latin American populations [43, 44], we estimate a substantially higher European contribution from the paternal side than from the maternal side in the ancestry lineage in Uruguay.

To analyse the contributions of maternal and paternal ancestry to infertility in a mixed population, we studied maternal and paternal ancestry in fertile and infertile men. Sperm parameters from infertile men were compared among haplogroups, looking for associations between ancestry and possible spermatogenic failure.

## Methods

### Subjects

To analyse the association between ancestry and infertility, we conducted a case control study. We recruited 120 infertile patients and 154 fertile men and typed both the Y chromosome and mitochondrial haplogroups. Infertile men were recruited from three different andrology centres that receive patients from the whole country.

The patients incorporated into this study consulted the infertility clinics during 2008–2011 because of couple infertility. After a complete clinical examination, semen analysis and Y chromosome microdeletion screening, all of the patients included were classified as having idiopathic infertility. The fertile group was composed of males from all over the country with at least one proved offspring.

All of the studies were anonymous, and the participants provided written informed consent. The School of Medicine-Universidad de la Republica Ethics Committee in Montevideo, Uruguay, approved the protocol.

### Semen analysis

Semen samples were obtained from patients by masturbation after sexual abstinence for at least 48 h. After liquefaction at 37 °C for 30 min, semen volume, pH, sperm concentration, vitality, motility, and normal morphology were determined. Routine semen analysis was performed in accordance with the fifth edition of the WHO guidelines [35]. Briefly, semen volume was measured with a graduated disposable pipette (BRAND®, Sigma- Aldrich, St Louis, MO, USA), and sperm concentration was assessed using a haemocytometer. Sperm motility was assessed by computer-assisted sperm analysis (SCA-Microoptics, Barcelona, Spain); semen aliquots (7 μl) were placed in CELL-VU disposable counting chambers (Millennium Sciences, Inc., New York, NY, USA). Two chambers were loaded, and a minimum of 500 spermatozoa was analysed in each specimen. The motility assessment was repeated twice (Pearson’s correlation r^2^ = 0.8, *p* < 0.0001), and the average value was calculated. Sperm motility was classified as follows: motility class a (fast progressive motility); b (slow progressive motility); c (all other patterns of motility with an absence of progression); and d (immotile sperm). In this study, sperm motility was assessed following the last WHO guidelines, which recommend using progressive motility (a + b), namely all spermatozoa that move actively, either linearly or in a large circle, regardless of their speed [35]. Sperm vitality was assessed using the eosin–nigrosin test in samples in which progressive motility was <50% [35].

Semen smears were stained by the Shorr method (IVD: In Vitro Diagnostic Medical Device Merck KGaA, Darmstadt, Germany) and were observed using a Nikon ECLIPSE E200 bright field microscope at 1000 magnification (oil immersion). Tygerberg’s strict criteria were used to assess sperm morphology in a minimum of 200 spermatozoa from at least 10 fields.

Based on the WHO nomenclature, the different forms of male factor infertility were distinguished as follows: low count or oligozoospermia (<15 million sperm cells per ml), total absence of sperm cells in the ejaculate or azoospermia; low progressive motility or asthenozoospermia (<32% a + b); and poor morphology or teratozoospermia (<4% normal sperm) [35].

### Y chromosome haplogrouping

We studied 154 and 120 DNA samples from fertile and infertile Uruguayan males, respectively, extracted from peripheral blood leukocytes, sperm or spittle by standard methods (DNeasy Blood and Tissue kit - Qiagen, Hilden, Germany). Each DNA sample was checked under an UV spectrophotometer (Biophotometa, Eppendorf, Hamburg, Germany).

**Fig. 1:**
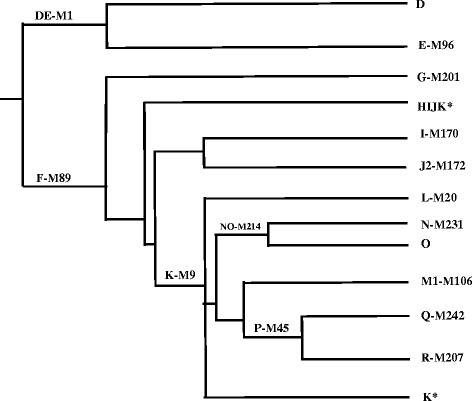
Phylogenetic tree constructed with 14 Y chromosome markers based on the YCC nomenclature. The Y haplogroups were classified according to the presence/absence of various binary markers. Each haplogroup and the diagnostic markers are indicated in the roots and in the ends of the branches. The DE* (putative D) or NO* (putative O) and HIJK* haplogroups were defined by the absence of the typed SNPs [9, 10]

The Y chromosome haplogroup analysis was performed hierarchically, as proposed by the YCC and the ISOGG (Fig. 1). To analyse the European, African and Native American contributions to the populations, a total of 14 biallelic Y chromosome markers were typed by assessing PCR product size (M1/YAP), assessing the absence/presence of PCR products using allele-specific primers (M89 and M9) or by real-time PCR and high-resolution-melting analysis. The primers used to type Y chromosome are shown in Additional file 1: Table S1.

High resolution melting (HRM) analysis was performed on the Rotor-Gene 6000™ real-time instrument (Corbett Life Science, Sydney, Australia) with Eva Green, a saturating dye technology (Type-it HRM PCR Kit, Qiagen, Hilden, Germany).

### Mitochondrial DNA ancestry informative markers

Samples were analysed and assigned to major mtDNA haplogroups through the analysis of control region sequences. As a first approach, hypervariable region I (HVRI, nucleotide positions [np] 16,024–16,400) following the revised Cambridge Reference Sequence [45] was amplified using primers 15,996 F [46] and 11R [47]. The amplification products were verified by electrophoresis on 2% agarose gel with ethidium bromide staining. PCR products for sequencing were purified using silica spin columns, and sequencing was performed by an external service provider (Macrogen Inc., Seoul, South Korea) using the same primers as those employed for PCR. Sequences were examined using Chromas software (Technelysium Pty Ltd.), version 2.01, and aligned using Genedoc software, version 2.7.000 [48]. Haplogroup definition was established using HaploGrep [49], based on Phylotree tree Build, version 17 [20]. In those cases in which the major haplogroup assignment was uncertain based on HVRI alone, hypervariable region II (HVRII, np 44–340) was amplified using primers 29F [46] and 397R [50] and was sequenced to obtain greater resolution.

### Statistical analysis

The difference in haplogroup distribution between cases and control samples was statistically tested using Fisher’s exact test on contingency tables for single haplogroups after Bonferroni’s correction.

The mean ± standard error of the mean (SE) of sperm parameters were determined in each Y chromosome haplogroup. D’Agostino and Pearson’s normality test was used to evaluate the normal distribution of the examined variables. One-way analysis of variance was used or the Kruskal–Wallis test when normality tests failed. Tukey’s test was applied to determine differences between groups. After dividing the groups according to cut-off values recommended by the WHO [35], logistic regression analysis was applied to identify associations between haplogroups and main seminal parameters. The analysis was performed with Epi Info 2000 software (Center for Disease Control and Prevention, Atlanta, GA, USA).

Statistical significance was indicated by *p* values < 0.05.

## Results

### Semen analysis

The mean ± SE of semen volume from the 120 infertile men was 3.6±0.25 (ranging from 1 to 9 ml). Three of them presented semen volumes less than normal values (<1.5 ml). Semen samples presented pH values between 7.5 and 8.2 (mean 7.9±0.4), and sperm vitality ranged between 64% and 96% (mean 90±1.7), indicating that both characteristics were within normal ranges (normal pH of semen is considered between 7.2 to 8.2, and the lower reference limit for vitality is 58%) [35].

Semen analysis of the infertile men showed oligozoospermia in 60% of cases with a mean sperm concentration of 3,524,111± 643,000 sperm cells per ml (ranging from 50,000 to 12,976,000 sperm cells per ml). Azoospermia was noted in 18% of 120 infertile men. With respect to sperm motility, 38% of the 120 infertile men showed values less than normal according to the WHO guidelines. Of the infertile men, 65% exhibited percentages of normal sperm morphology less than reference values (<4%).

The Y chromosome microdeletion screening showed a single positive case of *AZFc*.

### Y chromosome population analysis

The Uruguayan fertile and infertile men belonged to the seven main haplogroups that represents the main ancestry of the Uruguayan population: A-B, E, R, F(xK) (haplogroup F with no K individuals), Q, DE, and K(xP) (haplogroup K with no haplogroup P individuals). The hierarchical analysis determined more detailed subclade distributions of the F(xK) and K(xP) haplogroups (Table 1).

**Table 1:** Distribution of Y chromosome haplogroups in fertile and infertile men from Uruguay

Ancestry	Main haplogroup	Subclade	Fertile men (n)	Infertile men (n)
African	A-B		1	0
African-European	E		15	16
European	R		87	55
Eurasian	^a^F(xK)		39	38
		G	7	10
		F(xGIJ2K)	9	11
		I	11	11
		J2	8	5
Native American	Q		5	0
Asian	DE		0	1
Eurasian	K(xP)		4	7
		L	1	2
		M1	0	1
		N	3	2
		K(xLM1NOP)	0	2

Fisher’s exact probability test of the distributions of 7 haplogroups and their 13 subclades between fertile and infertile men; no differences were found (*p* > 0.05)

^a^Note that 4 fertile and 1 infertile men were only defined by the main haplogroup (see text)

When we compared haplogroup frequency among the fertile and infertile men, we found no significant differences between haplogroup distributions. R and F(xK) were the most dominant haplogroups among the whole study population, followed by haplogroup E. No individuals belonging to haplogroup Q were found in the infertile group, indicating that paternal Native American ancestry was not present at all among the cases.

Regarding subclade frequency, we did not find significant differences in the distribution between fertile and infertile men (Table 1).

### Mitochondrial DNA ancestry

The analysis of mtDNA revealed that the contributions from the three parental groups were 6.6% African, 24% Native American and 69.3% European. Among the controls, 3.1% of individuals carried an African haplogroup, 26.5% a Native American haplogroup, and 70.4% a European haplogroup, whereas among the cases, the respective figures were 7.5%, 27.4% and 65.1% (Table 2). The case and control groups were not different from each other (*p* = 0.35). A detailed distribution of each mitochondrial haplogroup analysed is also shown in Table 2.

**Table 2:** Mitochondrial DNA haplogroups in fertile and infertile men from Uruguay

Ancestry	Haplogroup	Fertile men (n)	Infertile men (n)
Native American			
	A	4	8
	B	5	8
	C	15	9
	D	2	4
European			
	H	34	42
	I	1	2
	J	7	6
	K	7	3
	N	1	2
	T	5	1
	U	14	12
	X	0	1
African	L	3	8

Fisher’s exact probability test of the distribution of the three ancestry groups and the 13 haplogroups between fertile and infertile men (*p* > 0.05)

### Correlations between ancestry and semen parameters

When semen parameters were compared between different groups following the WHO guidelines [35], men from Y chromosome haplogroup F(xK) had more risk (*p* < 0.01) of presenting normal sperm morphology less than 4% than men from other groups (Table 3). Mitochondrial ancestry was not associated with sperm morphology (Table 4). The other semen parameters were similar in all of the mitochondrial and Y chromosome haplogroups (Tables 3 and 4), including semen volume, pH and vitality (data not shown).

**Table 3:** Association analysis between Y chromosome haplogroups and sperm characteristics in infertile men

	Haplogroup E	Haplogroup F(xK)	Haplogroup D	Haplogroup K(xP)	Haplogroup R	ND
Semen analysis^a^						
	n (%)	n (%)	n (%)	n (%)	n (%)	n (%)
Sperm count						
< 15 × 10^6^/ml	9 (56.2)	26 (69.2)	0 (0.0)	5 (71.4)	36 (65.5)	2 (66.7)
> 15 × 10^6^/ml	7 (43.8)	12 (30.8)	1 (100)	2 (28.6)	19 (34.5)	1 (33.3)
Progressive Motility						
< 32%	6 (37.5)	14 (38.5)	1 (100)	2 (28.6)	29 (52.7)	1 (33.3)
> 32%	10 (62.5)	24 (61.5)	0 (0.0)	5 (71.4)	26 (47.3)	2 (66.7)
Morphology						
< 4%	10 (62.5)	33 (86.8)*	0 (0.0)	4 (57.1)	35 (63.6)	2 (66.7)
≥ 4%	6 (37.5)	5 (13.2)	1 (100)	3 (42.9)	20 (36.4)	1 (33.3)

Logistic regression analysis of haplogroup F(xK) vs. other haplogroups; *p = 0.003; odds ratio 5.5; 95% confidence interval (1.7–17.4)

^a^Semen analysis according to WHO [35]

**Table 4:** Association analysis between mitochondrial haplogroups and sperm characteristics in infertile men

	Haplogroup H,I,J,K,N,T,U	Haplogroup A,B,C,D	Haplogroup L	ND
Semen analysis^a^				
	n (%)	n (%)	n (%)	n (%)
Sperm count				
< 15 × 10^6^/ml	46 (66.7)	14 (50.0)	5 (62.3)	7 (35.3)
> 15 × 10^6^/ml	23 (33.3)	14 (50.0)	3 (37.8)	8 (64.7)
Progressive Motility				
< 32%	30 (43.5)	10 (36.4)	2 (25.0)	4 (33.3)
> 32%	39 (56.6)	18 (64.3)	6 (75.0)	11 (66.7)
Morphology				
< 4%	46 (66.7)	16 (61.5)	6 (75.0)	6 (35.3)
≥ 4%	23 (66.3)	10 (38.5)	2 (25.0)	11 (64.7)

Logistic regression analysis. No differences were found between haplogroups

^a^Semen analysis according to the WHO [37]

The mean ± standard error (SE) of normal morphology of sperm from men belonging to Y chromosome haplogroup F(xK) was 2.5 ± 0.3, which was significantly different from men in group R (4.7 ± 0.7) and lower than in the other groups; E (3.7 ± 1.1) and K(xP) (3.6 ± 1.3), according to ANOVA and Tukey post-test (*p* < 0.05) (Table 5).

**Table 5:** Mean ± standard error of sperm characteristics in infertile men

	Y Chromosome Haplogroups				Mitochondrial Haplogroups		
	E	F(xK)	K(xP)	R	H,I,J,K,N,T,U,X	A,B,C,D	L
Sperm count (10^6^/ml)	47.2 ± 1.7	20.2 ± 5.1	18.3 ± 10.1	29.1 ± 7.7	21.0 ± 5	39.4 ± 9.7	60.6 ± 33.1
Progressive Motility	34.3 ± 6.6	36.8 ± 3.4	45.6 ± 11.3	35.3 ± 3.9	34.1 ± 2.9	39.9 ± 5	47.9 ± 9.7
Morphology	3.7 ± 1.1	2.5 ± 0.3*	3.6 ± 1.3	4.7 ± 0.7*	3.3 ± 0.5	4.5 ± 1	3.3 ± 1.5

*p < 0.05 after ANOVA and Tukey’s multiple comparisons test between Y chromosome haplogroups

Regarding sperm parameters, no significant differences were found among haplogroups G, F(xGIJ2K), I, and J2 from F(xK) (Additional file 2: Table S2) or among L, M1, and N from K(xP) individuals (data not shown).

The means ± SEs of sperm morphology were similar among men with European, Native American and African maternal ancestry (Table 5).

Differences between the means ± SEs of sperm count and progressive motility were not statistically significant either between Y chromosome haplogroups or between mtDNA haplogroups (Table 5).

## Discussion

Although the idea that ethnic differences can impact reproduction has begun to be recognized in the literature [51–53], only a limited number of studies exist that have investigated either paternal or maternal ancestry in male fertility [14, 17–19, 25, 26, 54, 55]. None of the mentioned studies combined maternal and paternal origins, unravelling the possible impact of each parent’s ancestry in the development of male infertility.

In this study, we analysed the paternal and maternal ancestry of fertile and infertile men. The haplogroup distribution observed confirmed the previously characterized admixture attributes of the Uruguayan population [37, 42, 56, 57]. Moreover, indistinctly, fertile and infertile men presented much higher contributions of European origin from the paternal side than form the maternal side, an observation that has already been reported in the general population without considering their fertility potential [41, 42] .

Neither maternal nor paternal ancestry presented differences between the cases and controls.

The analysis of the infertile men’s sperm parameters resulted in some remarkable observations. We did find a higher risk of having spermiogram abnormalities in men belonging to particular Y chromosome haplogroups. In contrast, none of the sperm parameters studied was associated with any particular mtDNA maternal haplogroup. In contrast to what was previously reported, we did not find associations between maternal ancestry and sperm motility [26, 27].

Men belonging to haplogroup F(xK) presented a higher risk of having normal sperm morphology less than normal values [35] than men from other haplogroups.

The factors that produce abnormal sperm can be diverse and difficult to elucidate. The genes that produce abnormal sperm are partially known in cases of monomorphic hereditary teratozoospermia [58], but genetic causes of idiopathic teratozoospermia are unknown. Some genes that determine sperm morphology might be inherited together with the region that determines the F(xK) haplogroup.

The role of the Y chromosome in male reproductive failure is indisputable since it contains the master gene of testis development (SRY) and a number of genes with specific roles in spermatogenesis [12]. Moreover, the definition of Y chromosome haplogroups in patients with altered spermiograms could be the first step towards the identification of patients with Y chromosome-related factors leading to spermatogenic failure. Apart from classical microdeletions, other Y chromosome anomalies can cause spermatogenic failure, e.g., partial gene copy deletions of multicopy genes, inversions, and rearrangements in noncoding regions, but with possible functional effects on gene expression. These types of alterations can segregate within certain backgrounds; therefore, the determination of Y chromosome haplogroups that associate with infertility could identify ethnic groups at risk [12]. For example, in the Italian population, the testis-specific protein Y-encoded gene *(TSPY)* copies were lower in haplogroup P, and a low copy number of *TSPY* was associated with infertility [59]. Microdeletions were also more frequently found in particular haplogroups, depending on the region where the study was performed [60, 61]. In Chile, the Amerindian Q-M3 haplogroup seemed to be correlated with Y micro-deleted chromosomes [60]. In Italy and Spain, haplogroup P and its subclade R or haplogroup E [62] showed associations with micro-deletions demonstrating the heterogeneity and relevance of micro-geographic differences in male populations. Relative to our study, SNPs that determine subclades such as G, I, H, and J2 included in haplogroup F(xK) are in close proximity to some genes that could be associated with infertility [9]. For example, rs2032597 (M170) and rs2032636 (M201) are SNPs close to ubiquitin-specific protease 9, Y-linked (*USP9Y*). *USP9Y* is localized in a region of the Y chromosome known to be associated with infertility (*AZFa*) [63].

USP9Y’s precise function in the cell is unknown. On the one hand, *USP9Y* deletion is compatible with normal spermatogenesis and fertility [64]. On the other hand, its loss can disrupt spermatogenesis to varying degrees [64, 65]. It has been postulated that the phenotype associated with the loss of *USP9Y* varies according to the genetic or other background of the carrier, but the relevant modifying factors remain unknown [66].

Caution must be taken when comparing between infertile and control men in search of genetic susceptibility factors related to the Y chromosome [67]. The Y chromosome is more complex for association studies than the remainder of the genome because of population stratification, and it requires unusual levels of confirmation. Precise geographical matching is essential, and replication in an independent sample has also been recommended [67]. The relatively small population and territory of Uruguay permitted our study to meet the first requirement. Furthermore, the similar distribution of the Y and mtDNA haplogroups between the cases and controls reinforces the importance of the association that we found between semen parameters and a specific haplogroup. A weakness of our study was the absence of an independent analysis of another group of infertile men of haplogroup F(xK), so our data should be interpreted as exploratory, with the intent of identifying possible guides to understand the genetics of male infertility.

Since infertility is a multifactorial disease, we cannot ignore the possibility that other factors, such as access to infertility clinics, and environmental factors (diet, cultural, social, etc.) can account for some of the differences with other studies. In this study, we focused on male factors, attempting to discard the cases in which a clear cause of female infertility was found. Nevertheless, since infertility is considered a couple disease, female genetic background can also be considered in future studies. An interesting finding was the absence of a Native American paternal contribution in the infertile population, likely because of the low representation of this ancestry in the Uruguayan population.

Finally, analysis of mtDNA and non-recombinant region Y variations in human genomes has provided numerous important insights into the maternal and paternal histories of the migrations of human populations [68, 69]; however, very few studies have analysed the possible association of these markers with fertility. Our data supported the idea that paternal, but not maternal, ancestry is related to male fertility. It is tempting to speculate that the major contribution of European origin from the paternal side (lower Y chromosome diversity, compared to mitochondrial diversity) of the Uruguayan admixed population might have exerted selective pressure on the population by modifying fertility potential when men moved to South American countries. Further studies are necessary to assess the role of the demographic process in and the population contributions to male fertility, especially in admixture populations such as Uruguayans.

## Conclusions

The data presented in this study confirmed the admixture characteristic of the Uruguayan population, showing that both fertile and infertile men had higher paternal than maternal European contributions. Neither maternal nor paternal ancestry differed between the two groups in the Uruguayan population. However, abnormal sperm morphology was more frequently found in infertile men from group F(xK), indicating that the Y chromosome from these men could be a target to look for genes that are related to sperm morphology.

In addition, the role of the demographic process in male fertility in mixed populations requires deeper analysis.

## Additional files

Additional file 1: Table S1. Primers for Y chromosome haplogroups analysis. (DOCX 72 kb)

Additional file 2: Table S2. Mean ± standard error of sperm characteristics in infertile men belonging to the G, F(xGIJ2K), I and J2 haplogroups. (DOCX 48 kb)

## Abbreviations

HRM: High resolution melting
HVR: Hypervariable region
ISOGG: International Society of Genetic Genealogy
MSY: Male-specific Y
mtDNA: Mitochondrial DNA
np: Nucleotide position
OXPHOS: Oxidative phosphorylation subunits
WHO: World Health Organization
YCC: Y Chromosome Consortium
