# Supplementary material for: Associations between male infertility and ancestry in South Americans: a case control study
Source: BMC Med Genet. 2017 Jul 26;18:78. doi: 10.1186/s12881-017-0438-z (PMC5530489; doi:10.1186/s12881-017-0438-z)
Supplement: Supplementary file 1 — Primers for Y chromosome haplogroups analysis. (DOCX 72 kb) [file 12881_2017_438_MOESM1_ESM.docx]

| **Supplementary Table 1. Primers for Y chromosome** **haplogroups analysis.** | | | | | |
| --- | --- | --- | --- | --- | --- |
| **Name** |  | **Sequence** |  | | **Size (bp)** |
|  |  |  |  | |  |
| M1 | 5´-CAGGGGAAGATAAAGAAATA-3´ | | | | 455 ( Alu+) |
|  | 5´-ACTGCTAAAAGGGGATGGAT- 3´ | | | | 150 (Alu-) |
|  |  |  |  | |  |
| M9 | 5´-GCCTAAGATGGTTGAATC- 3´ | | | | 276 |
|  | 5´-GCCTAAGATGGTTGAATG- 3´ | | | |  |
|  | 5´-CTCAAGCGTAAATGTACTGT- 3´ | | | |  |
|  |  | | | |  |
| M89 | 5´-TATATTTTACACATTTTTGGGCC- 3´ | | | | 365 |
|  | 5´-TCAGGCAAAGTGAGAGATA- 3´ | | | |  |
|  | 5´-TCAGGCAAAGTGAGAGATG– 3´ | | | |  |
|  |  | | | |  |
| M20 | 5’-GTACAGTTGGCCCTTTGTG-3’ | | | 109 | |
|  | 5’-CATGTTCAGTGCAAATGC-3’ | | |  | |
|  |  | | |  | |
| M45 | 5´-GGACTTTACGAACCAACC– 3´ | | | 116 | |
|  | 5´-ACTATCTCCTGGCCTGGAC– 3´ | | |  | |
|  |  | | |  | |
| M96 | 5´-ACCCACCCACTTTGTTGC– 3´ | | | 120 | |
|  | 5´-GTGAGCTGTGATGTGTAACTTG– 3 ´ | | |  | |
|  |  |  |  |  | |
| M106 | 5’-GGTCATCACCAAGCATAG-3’ | | | 91 | |
|  | 5’-ATACCTACCTTCCTTATTGTC-3’ | | |  | |
|  |  | | |  | |
| M170 | 5’-CTTTCAACATTTAAGACCAC-3’ | | | 117 | |
|  | 5’-GTGCATTATACAAAT TACTA-3’ | | |  | |
|  |  | | |  | |
| M172 | 5´-GCCTCTCAGTATCAACAGG- 3´ | | | 128 | |
|  | 5´-GGTACAGAGAAAGTTTGGAC- 3´ | | |  | |
|  |  | | |  | |
| M201 | 5’-GGCATAGTATCTTGTTCAAC-3’ | | | 100 | |
|  | 5’-AAT CCA GTATCAACTGAGG-3’ | | |  | |
|  |  | | |  | |
| M207 | 5´- GAAGTATCCCTGAAGAAGG –3´ | | | 116 | |
|  | 5´- CCTCTTGTTGGAAGATTATT –3´ | | |  | |
|  |  | | |  | |
| M214 | 5’-GCTGCTGATACAACACAC-3’ | | | 102 | |
|  | 5’-TGGGAGACAGTGTGAGAC -3’ | | |  | |
|  |  | | |  | |
| M231 | 5´-CAACATTTACTGTTTCTACTG C- 3´ | | | 108 | |
|  | 5´-ATCCAGTACAGCAAGTTTATT C- 3´ | | |  | |
|  |  | | |  | |
| M242 | 5´-CGGCATAGAAAGTTTGTG–3´ | | | 133 | |
|  | 5´-CTAGAACAACTCTGAAGCG–3 ´ | | |  | |
|  |  | | |  | |
