# Supplementary material for: Associations between male infertility and ancestry in South Americans: a case control study
Source: BMC Med Genet. 2017 Jul 26;18:78. doi: 10.1186/s12881-017-0438-z (PMC5530489; doi:10.1186/s12881-017-0438-z)
Supplement: Supplementary file 2 — Mean ± standard error of sperm characteristics in infertile men belonging to the G, F(xGIJ2K), I and J2 haplogroups. (DOCX 48 kb) [file 12881_2017_438_MOESM2_ESM.docx]

**Supplementary Table 2. Mean ± standard error of sperm characteristics in infertile men belonging to the G, F(xGIJ2K), I and J2 haplogroups**

| **Haplogroups** | **G** | **F(xGIJ2K)** | **I** | **J2** |
| --- | --- | --- | --- | --- |
| Sperm count (10^6^/ml) | 14.1±7.5 | 29.2±12.1 | 20.1 ±10.8 | 16.7±7.9 |
| Progressive Motility | 36.9±7.7 | 42.5±6.2 | 40.4 ± 4.0 | 40.2 ±7.7 |
| Morphology | 2.0±0.9 | 2.7±0.5 | 2.0±0.5 | 2.2±0.2 |

ANOVA Test among the G, F(xGIJ2K), I and J2 Y chromosome haplogroups. No differences were found between haplogroups (p>0.05).
